# Supplementary material for: Real-time imaging of rotation during synthesis by the replisome
Source: Sci Adv. 2026 Jan 1;12(1):eadx4089. doi: 10.1126/sciadv.adx4089 (PMC12757025; doi:10.1126/sciadv.adx4089)
Supplement: Supplementary file 1 — Figs. S1 to S17 Tables S1 and S2 Legends for movies S1 and S2 [file sciadv.adx4089_sm.pdf]

Science Advances
AAAS

Supplementary Materials for

Real-time imaging of rotation during synthesis by the replisome

Thomas M. Retzer *et al.*

Corresponding author: Karl E. Duderstadt, karl.duderstadt@tum.de

Sci. Adv. **12**, eadx4089 (2026)
DOI: 10.1126/sciadv.adx4089

The PDF file includes:

Figs. S1 to S17
Tables S1 and S2
Legends for movies S1 and S2

Other Supplementary Material for this manuscript includes the following:

Movies S1 and S2

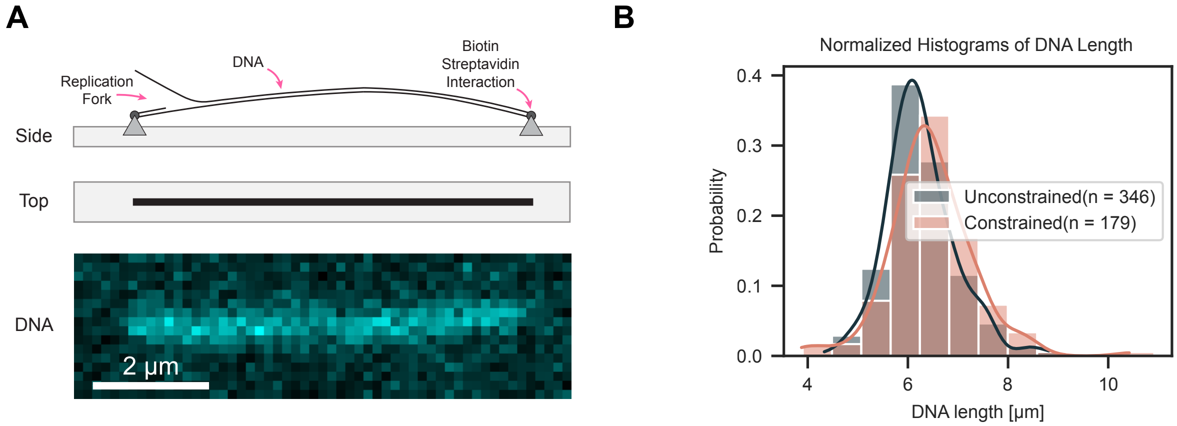

Fig. S1. Molecule length distributions. (**A**) DNA attachment in linear flow cell configuration. Cartoon indicates replication fork where replication is initiated. DNA is tethered to the surface utilizing a biotin streptavidin interaction. (**B**) Length Distribution for the two substrate types in µm. Number of molecules is indicated in the legend. Unconstrained and constrained are stretched to 68±7% and 69±9% (Mean ± SD), respectively.

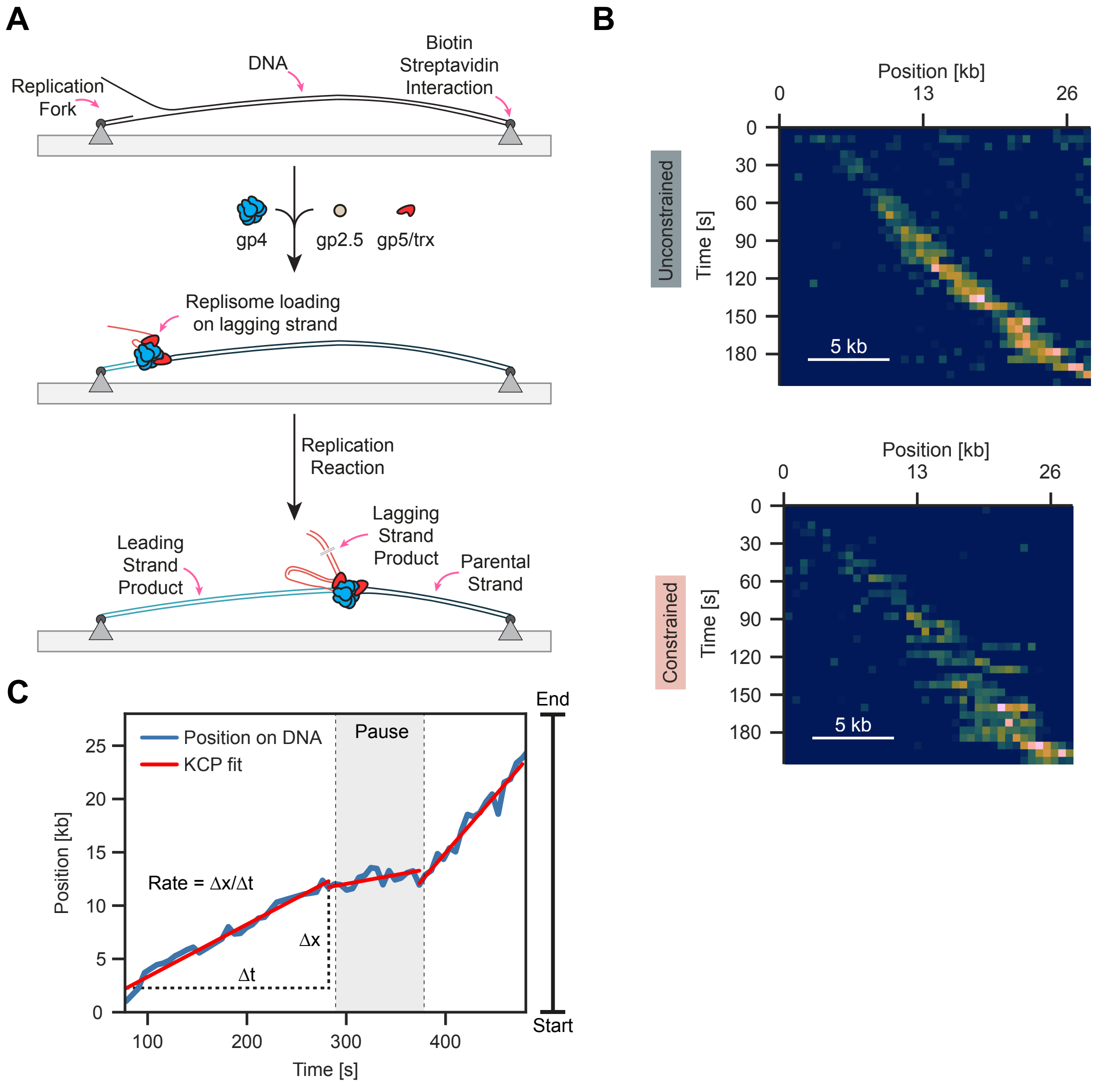

Fig. S2. Replication using a linear flow configuration. (**A**) Schematic of replication. DNA is tethered to the surface using biotin-streptavidin interactions. T7 replication components initiate replication on the preformed fork. Leading strand product forms behind the replisome while the lagging strand product forms a blob at the location of the replisome used to track progression. (**B**) Example kymographs for single-molecule replication on unconstrained and constrained molecules. (**C**) Single-molecule tracking of lagging-strand product resulting in a time trace containing replication rates and processivities.

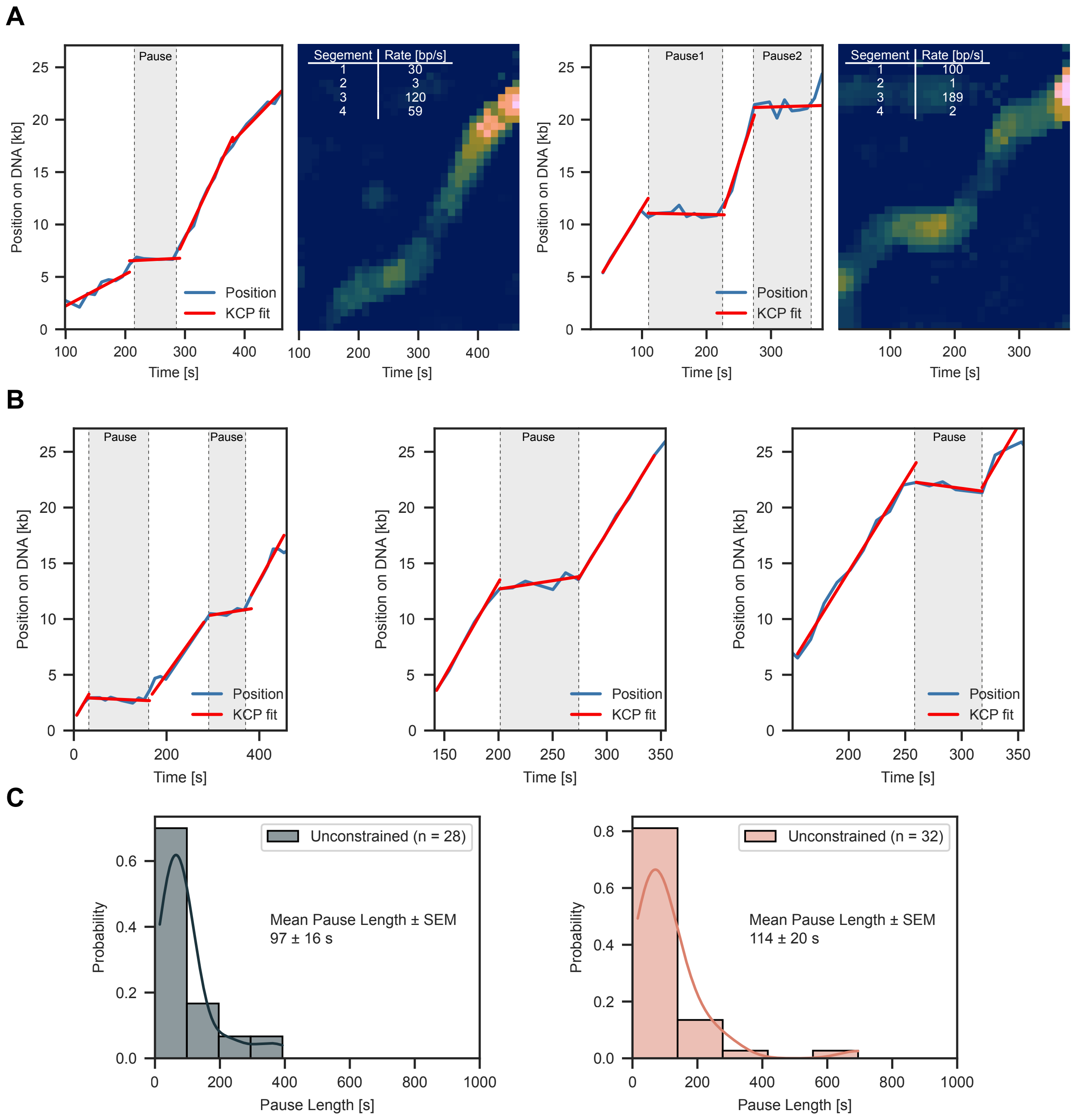

Fig. S3. Pausing behavior. (**A**) Example traces and kymographs showing single or double pausing events during replication. The table represents rates fitted using kinetic change point analysis. (**B**) Additional example traces for pausing. (**C**) Representative pause duration distribution for unconstrained and constrained molecules.

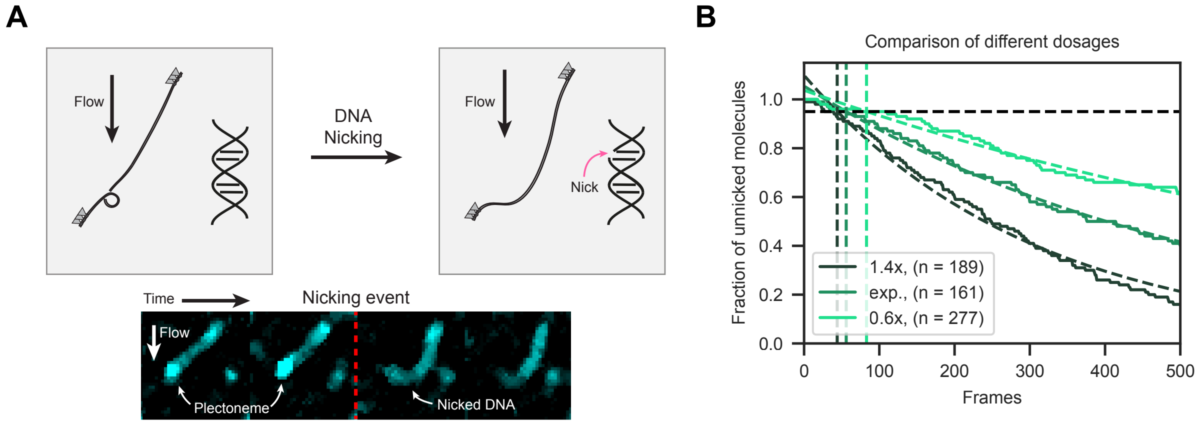

Fig. S4. Nick detection assay for quantification of laser-induced damage. (**A**) Concept of nicking assay to control for imaging condition introducing nicks in individual strands. DNA is supercoiled and upon nick introduction supercoils are relaxed and result in an increase in the overall size of the DNA shape. (**B**) Survival curve for unnicked molecules over time fit with an exponential decay curve. Three different laser settings were compared. 1.4 times and 0.6 times the laser setting of the experimental condition ('exp') are displayed.

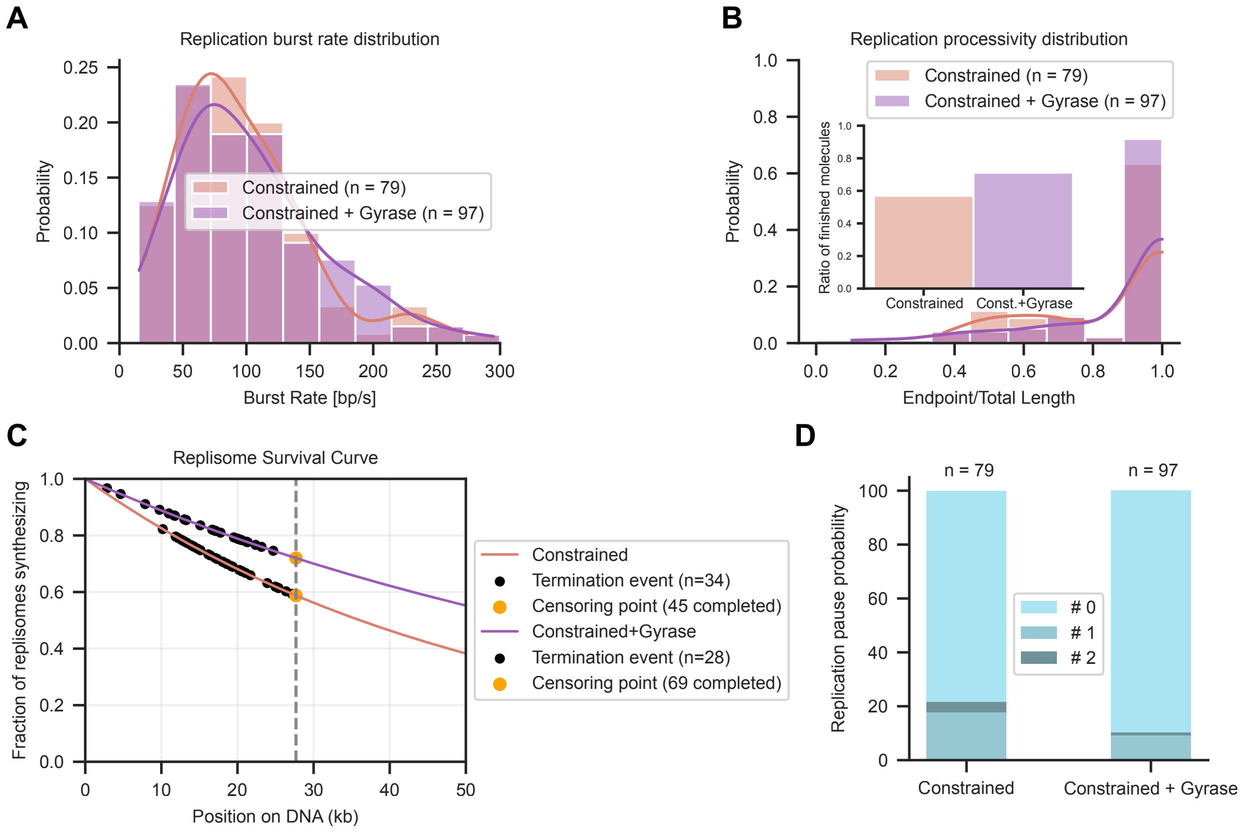

Fig. S5. Visualizing replisome progression under topological strain with DNA gyrase. (**A**) Replication burst rate distribution for constrained and constrained including DNA gyrase. (**B**) Replication processivity distribution for constrained and constrained including DNA gyrase. Inset displays the fraction of molecules that replicated to the end. (**C**) Replication processivity estimation using maximum likelihood estimation. (**D**) Pause probabilities for constrained and constrained including DNA gyrase.

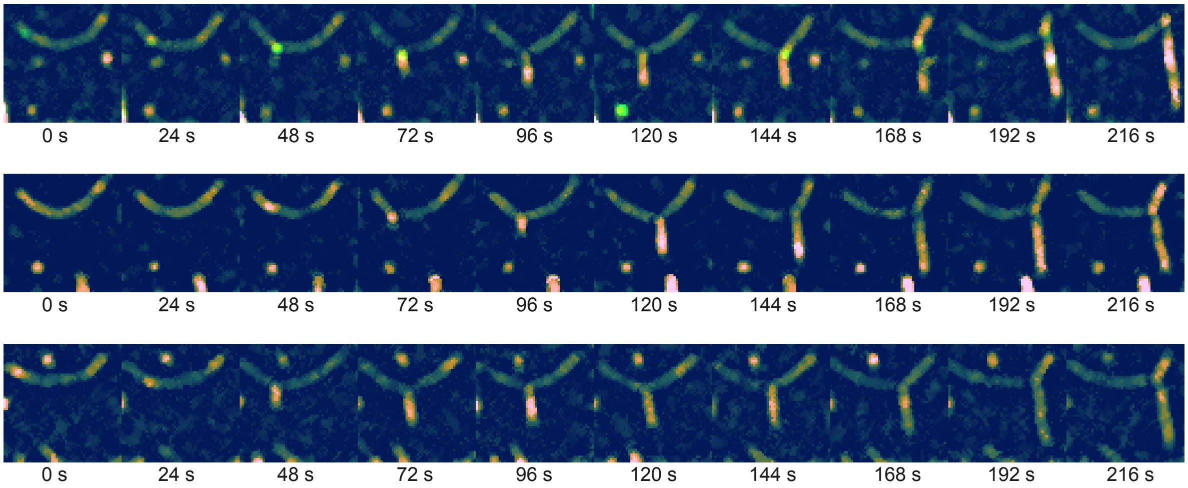

Fig. S6. Representative molecules for transverse flow imaging of DNA replication (unconstrained). Three representative montages for replication reactions with transverse flow for unconstrained molecules.

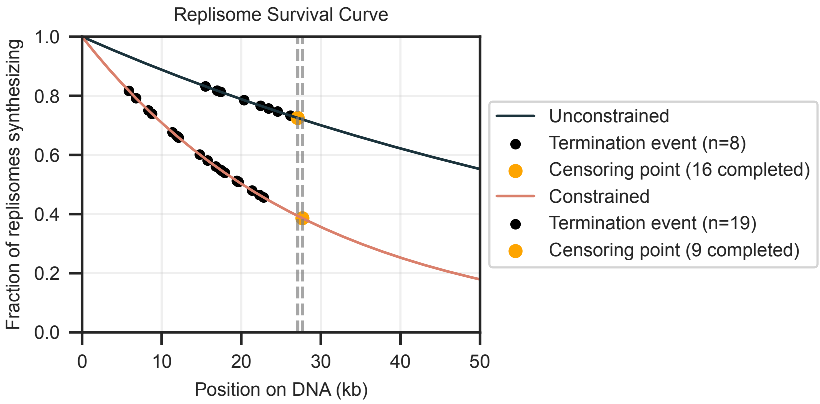

Fig. S7. Processivity for transverse flow. Replication processivity using maximum likelihood estimation.

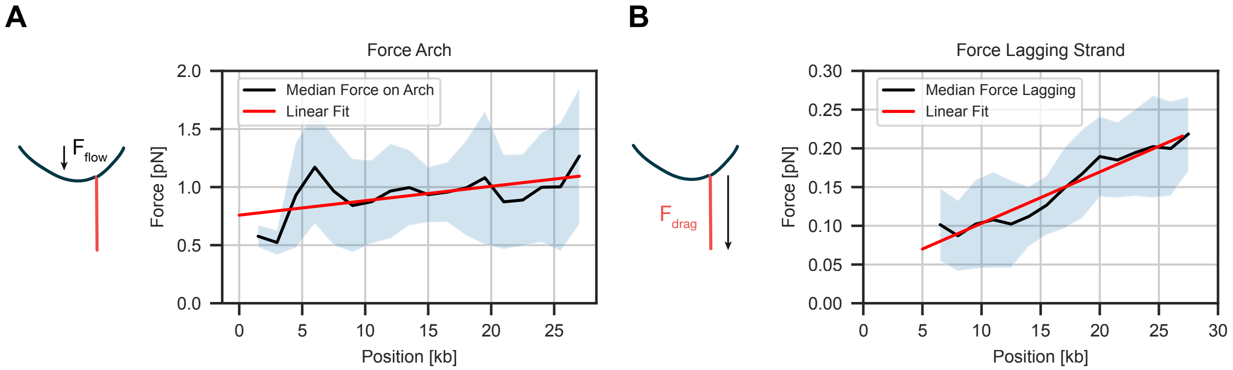

Fig. S8. Estimation of applied forces from transverse flow. (**A**) Force on the arch applied by the flow. Force was calculated comparing arch extension to the contour length of the DNA substrate. (**B**) Applied force on the lagging-strand product due to transverse flow as a function of product position. Worm-like chain model was used for force estimation and a linear fit was added. Standard error of the mean indicates error margin.

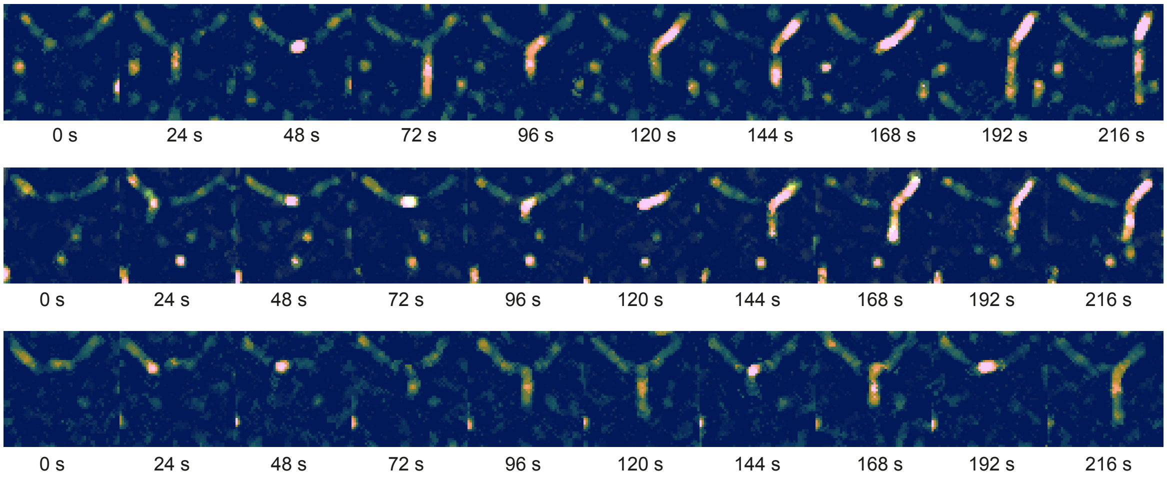

Fig. S9. Representative molecules for transverse flow imaging of DNA replication (constrained). Three representative montages for replication reactions with transverse flow for constrained molecules.

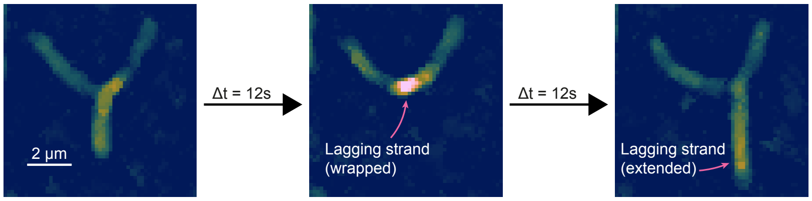

Fig. S10. Montage of time points showing fork rotation. Three consecutive time points first showing an elongated lagging strand product, followed by complete wrapping (DNA blob) and finally re-extension of the lagging-strand replication product.

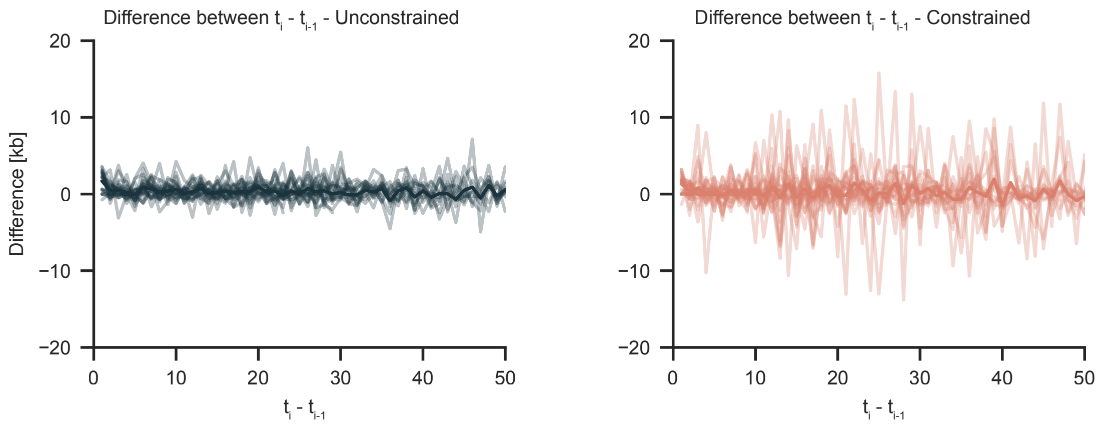

Fig. S11. Transformed lagging strand length. Transformation of lagging strand length over time (Fig. 4F) from non-stationary function to stationary function. To transform, the difference is taken for each point by subtracting the value from the previous timepoint.

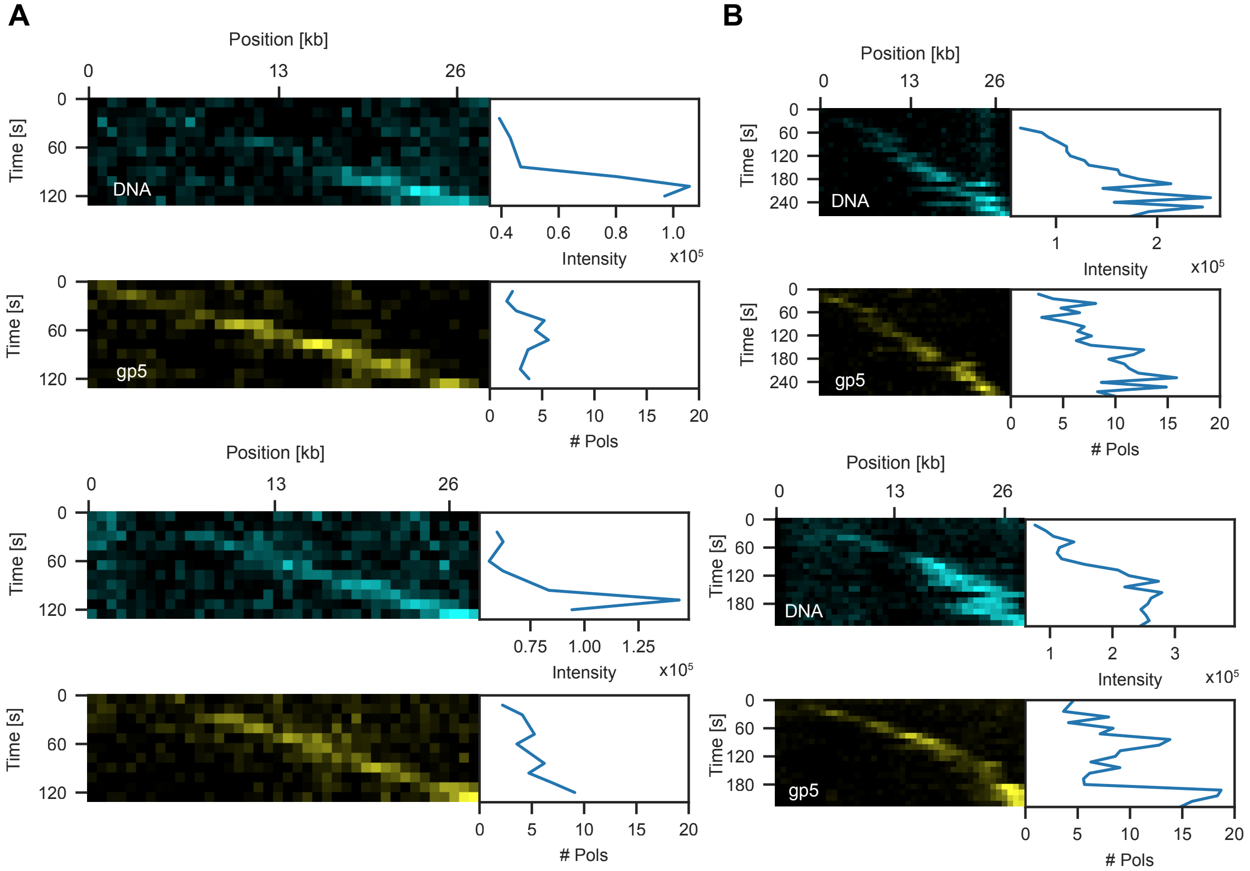

Fig. S12. Representative molecules for labeled polymerases during DNA replication. (**A**) DNA replication events in the absence of flow on unconstrained molecules. (**B**) DNA replication events in the absence of flow on constrained molecules. Stained DNA is displayed in cyan on the top and polymerase signal is displayed in yellow on the bottom.

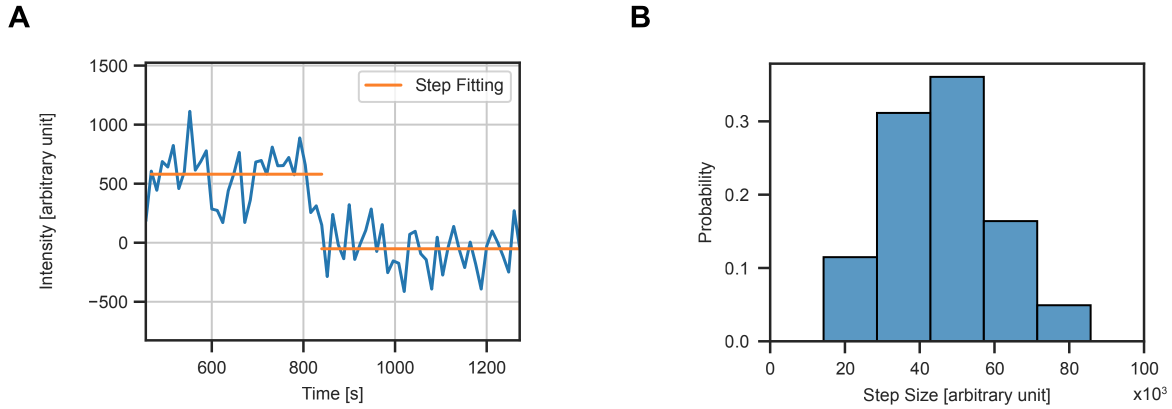

Fig. S13. Estimation of fluorescent signal of a single labeled polymerase. (**A**) Bleach step of a surface immobilized polymerase. Step was fitted using change point analysis. (**B**) Distribution of different step sizes for one single molecule dataset.

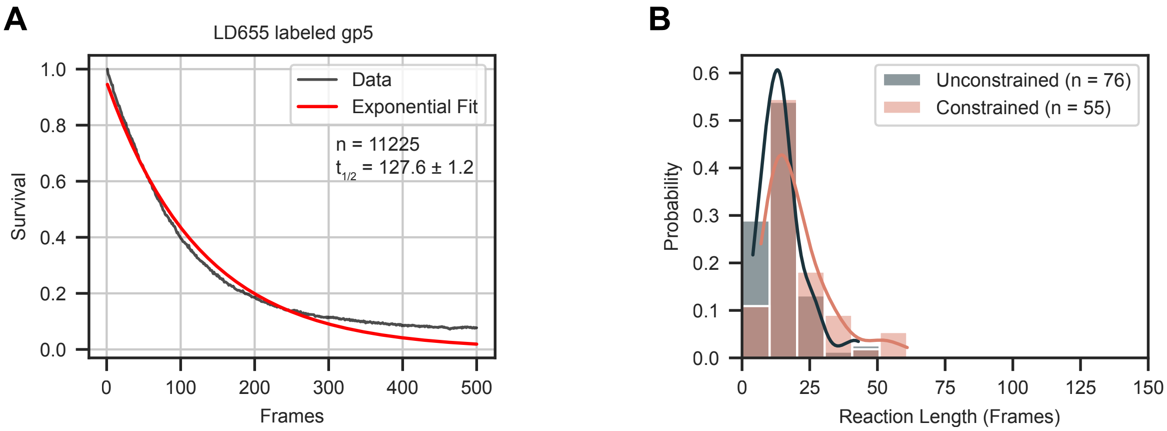

Fig. S14. Lifetime of single LD655 labeled gp5. (**A**) Lifetime of single, surface bound LD655 labelled gp5 dyes. These were used to determine the number of frames until half of the dyes bleached. Half-life shown as mean ± SD. (**B**) Distribution of reaction times for replication events for unconstrained (15 ± 1 frames, mean ± SEM) and constrained (21 ± 2 frames, mean ± SEM).

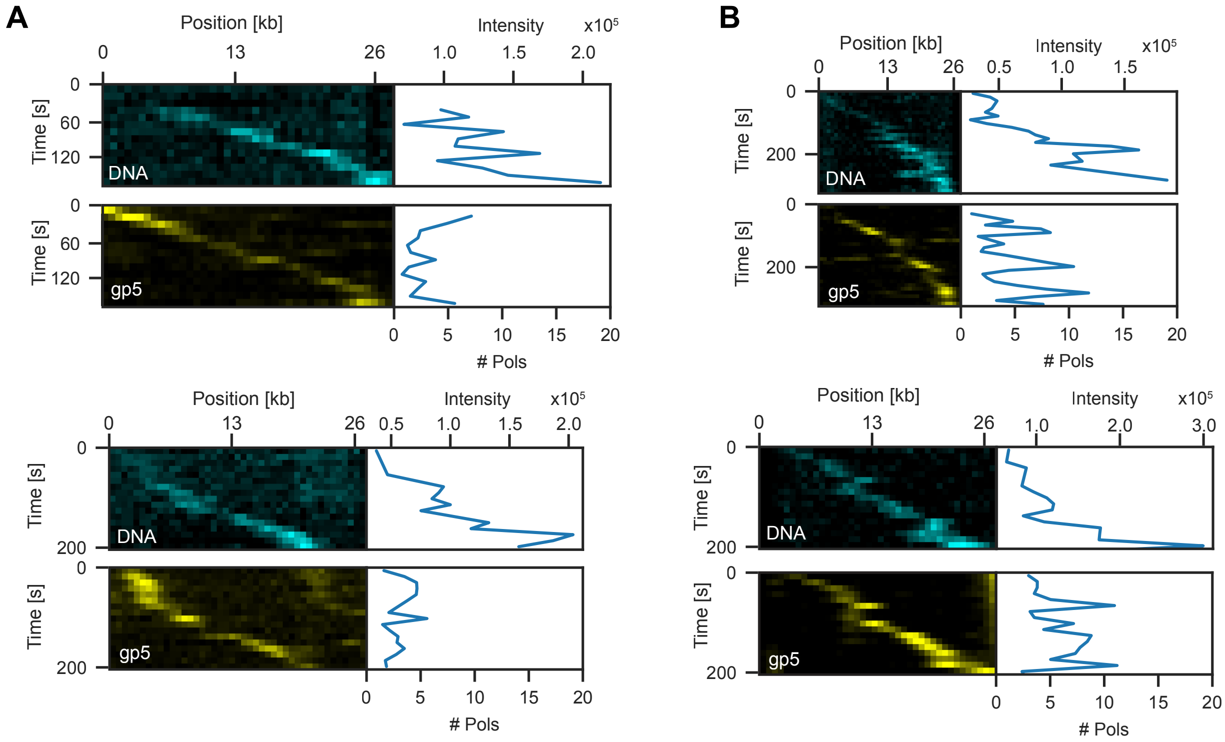

Fig. S15. Representative molecules for labelled polymerases during DNA replication under pre-assembly conditions with additional gp5. (**A**) DNA replication events in the absence of flow on unconstrained molecules. (**B**) DNA replication events in the absence of flow on constrained molecules. Stained DNA is displayed in cyan on the top and polymerase signal is displayed in yellow on the bottom.

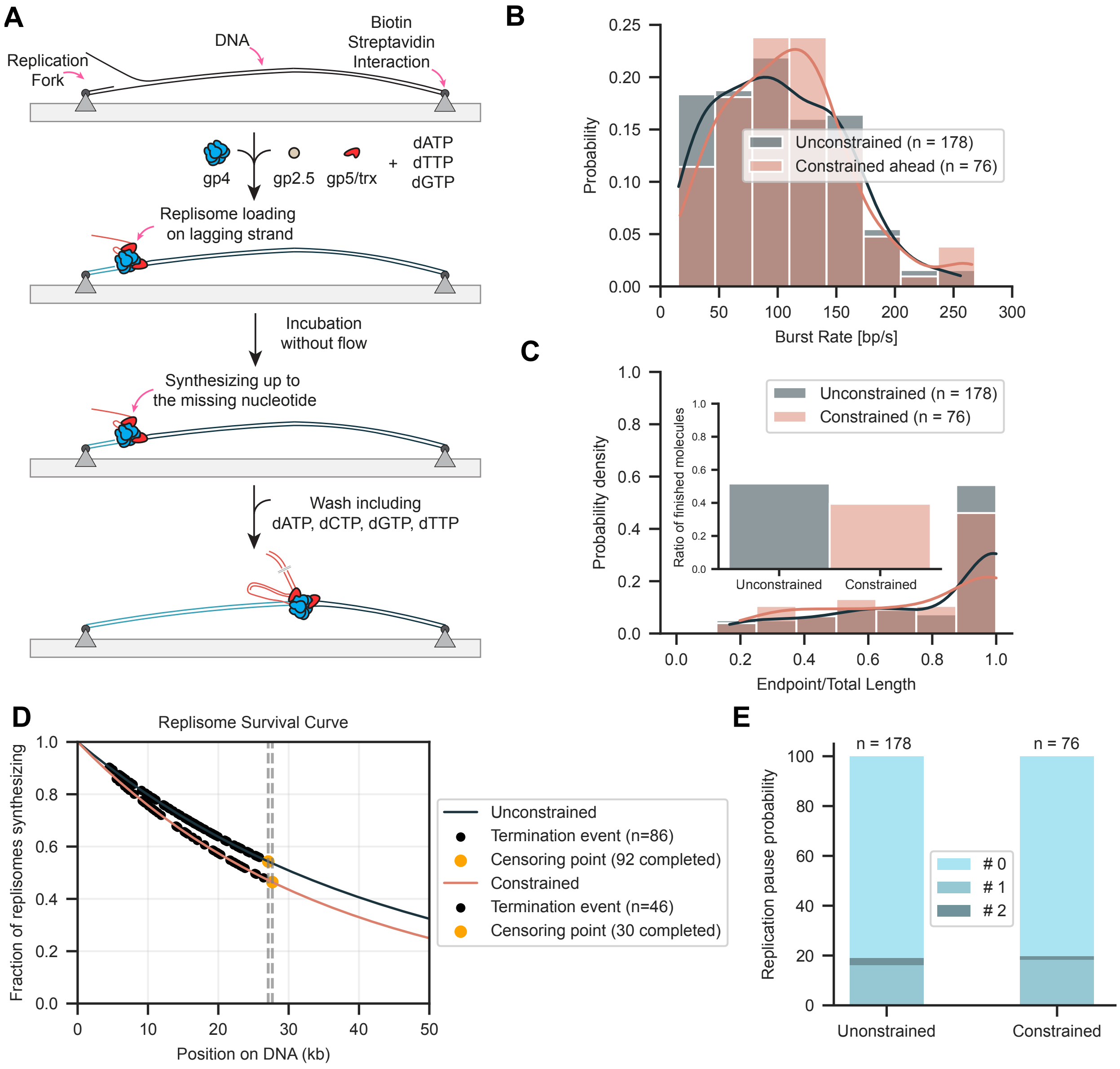

Fig. S16. Replication using pre-assembled replisomes. (**A**) Schematic of replication assay using pre-assembly condition. (**B**) Replication burst rate distribution for unconstrained and constrained molecules. (**C**) Replication processivity distribution for unconstrained and constrained molecules. Inset displays the fraction of molecules that replicated to the end. (**D**) Replication processivity estimation using maximum likelihood estimation (**E**) Pause probabilities for unconstrained and constrained molecules.

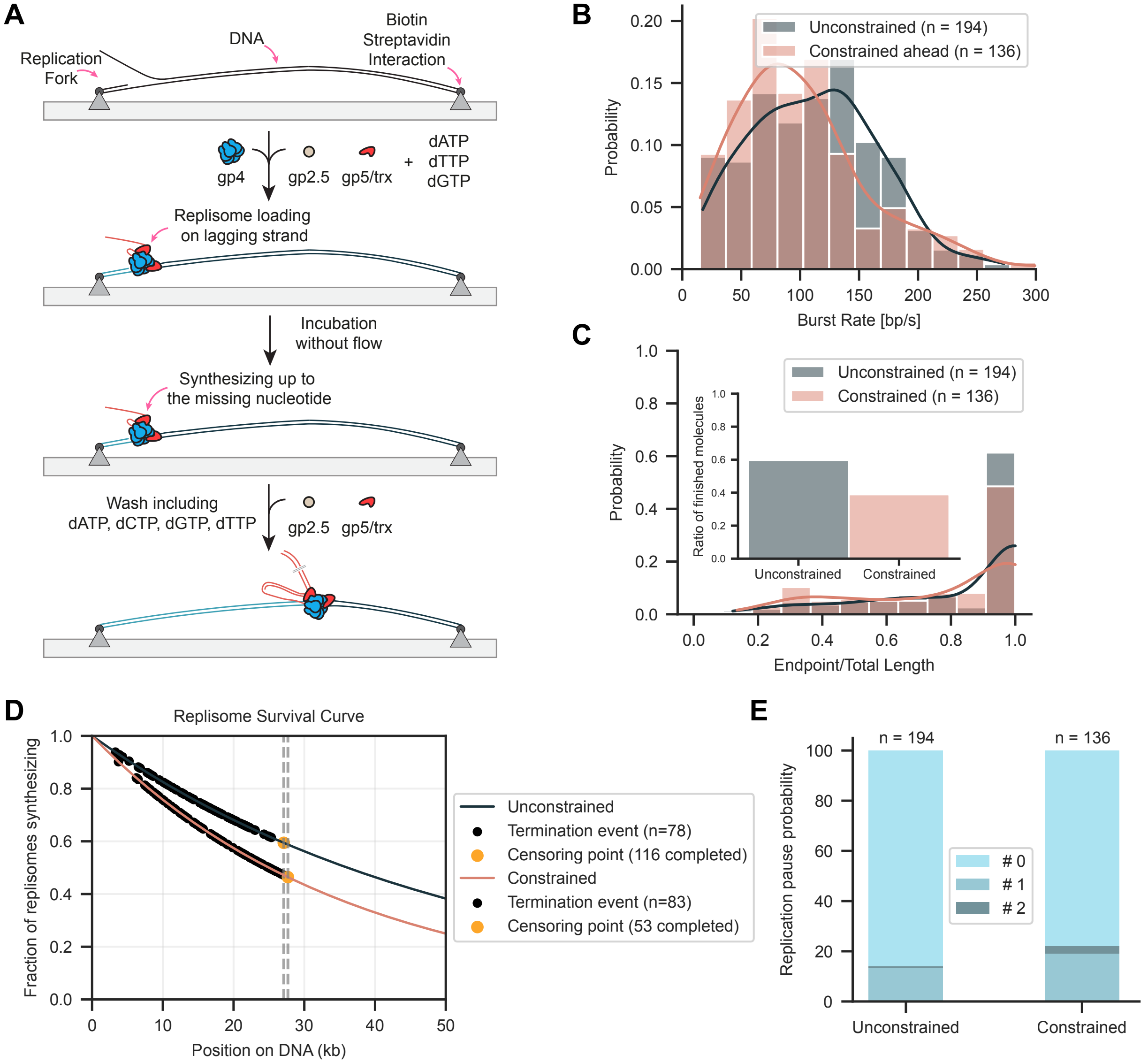

Fig. S17. Pre-assembly condition with additional gp5 during replication. (**A**) Schematic of replication assay under pre-assembly conditions with additional gp5 during replication. (**B**) Replication burst rate distribution for unconstrained and constrained molecules. (**C**) Replication processivity distribution for unconstrained and constrained molecules. Inset displays the fraction of molecules that replicated to the end. (**D**) Replication processivity estimation using maximum likelihood estimation. (**E**) Pause probabilities for unconstrained and constrained molecules.

Experimental Type	Modification	DNA Type	Mean Burst Rate [bp/s] Mean±SEM	Processivity Estimation [kb] Mean (95% CI)	Fraction of Fully replicated molecules [%]	Number of molecules	Figure Ref.
Linear		Uncon.	99 ± 3	117 (102,137)	75	186	2
		Con.	96 ± 5	52 (43, 67)	57	79	2
	Pre-assembly	Uncon.	101 ± 3	44 (39, 52)	52	178	S16
	Pre-assembly	Con.	108 ± 5	36 (29, 47)	39	76	S16
	Pre-assembly+gp5	Uncon.	112 ± 3	52 (46, 61)	60	194	S17
	Pre-assembly+gp5	Con.	100 ± 4	36 (31, 43)	39	136	S17
	Gyrase (5 nM)	Con.	103 ± 5	84 (70, 105)	71	97	S5
Transverse		Uncon. (leading)	97 ± 7	84 (60, 140)	67	24	3,4,S7
		Con. (leading)	98 ± 9	29 (21, 46)	32	28	4,S7
Pausing							
Experimental Type	Modification	DNA Type	0 Pause	1 Pause	2 Pause	Number of molecules	
Linear		Uncon.	0.88	0.12	0.01	186	2
		Con.	0.78	0.18	0.04	79	2
	Pre-assembly	Uncon.	0.81	0.16	0.03	178	S16
	Pre-assembly	Con.	0.80	0.18	0.01	76	S16
	Pre-assembly+gp5	Uncon.	0.86	0.13	0.01	194	S17
	Pre-assembly+gp5	Con.	0.78	0.19	0.03	136	S17
	Gyrase (5 nM)	Con.	0.9	0.09	0.01	97	S5

Blob size					
Experimental Type		DNA Type	Blob size [µm] Mean±SD	Number of molecules	
Linear		Uncon.	1.1 ± 0.2	114	2
		Con.	1.5 ± 0.4	52	2
Number of Polymerases					
Experimental Type	Modification	DNA Type	Mean number of polymerases Mean±SEM	Number of molecules	
Linear		Uncon.	3.75 ± 0.08	76	5
		Con.	11.40 ± 0.22	55	5
	Pre-assembly	Uncon.	2.07 ± 0.04	93	5
	Pre-assembly	Con.	2.92 ± 0.08	55	5
	Pre-assembly+gp5	Uncon.	3.61 ± 0.05	165	5
	Pre-assembly+gp5	Con.	4.91 ± 0.09	97	5

Table S1. Summary statistics from single-molecule microscopy experiments organized by experimental conditions and assay modification, reporting rates, processivities, fraction fully replicated, blob size analysis, number of polymerases and molecule numbers.

Name	Sequence
Oligo1	TATTAGCGGCCGCGATTGTTCTTTATTCATTTT
Oligo2	CCCATCGGAAAACTCCTGCTTTAGC
Oligo3	TTACCGCATACCAATAACGCTTCAC
Oligo4	GAAACTCAACATCGTCATCAAACGC
Oligo5	[BIO] AGGTCGCCGCCC
Oligo6	[PHO] TCGAGGGCGGCGACCT
Oligo7	[PHO] CTAGAGACAGCAAGTTGGACAATCCATCTCGTTCTATCACTAATGCAGGGAGGATTTCAGATATGGCAACTAGTATGCCG
Oligo8	TTTTTTTTTTTTTTTTTTTTTTTTTTTTTTATGGATTGTCCAACTTGCTGTCT
Oligo9	[BIOTEG] TTTTTTTTTTCGGCATACTAGTTGCCATATCTGAAATCCTCCCTGC

Table S2. List of sequences used to create replication substrates for single-molecule experiments.

Movie S1. Representative unconstrained molecule during DNA replication imaged using transverse flow. The individual leading, lagging, and parental strands are all spatially resolved. The lagging-strand product is seen growing out from along the arch and extending downward due to applied flow. Intensity is color-coded using the Batlow LUT. Time is displayed as minutes and seconds in the format (mm:ss).

Movie S2. Representative constrained molecule during DNA replication imaged using transverse flow. The lagging-strand product is seen wrapping around the arch during ongoing DNA synthesis due to fork rotation. Transient downward extension events are observed in between bursts of fork rotation. Intensity is color-coded using the Batlow LUT. Time is displayed as minutes and seconds in the format (mm:ss).
